# CD16 and CD57 expressing gamma delta T cells in acute HIV-1 infection are associated with the development of neutralization breadth

**DOI:** 10.1371/journal.ppat.1012916

**Published:** 2025-01-31

**Authors:** Gina L. Griffith, Kawthar Machmach, Ningbo Jian, Dohoon Kim, Margaret C. Costanzo, Matthew Creegan, Isabella Swafford, Gautam Kundu, Lauren Yum, Jessica S. Bolton, Lauren Smith, Bonnie M. Slike, Elke S. Bergmann-Leitner, Rasmi Thomas, Nelson L. Michael, Julie A. Ake, Leigh Anne Eller, Merlin L. Robb, Samantha M. Townsley, Shelly J. Krebs, Dominic Paquin-Proulx, for the RV217 Study Group

**Affiliations:** 1 US Military HIV Research Program, Center for Infectious Disease Research, Walter Reed Army Institute of Research, Silver Spring, Maryland, United States of America; 2 Henry M. Jackson Foundation for the Advancement of Military Medicine, Bethesda, Maryland, United States of America; 3 Biologics Research and Development, Walter Reed Army Institute of Research, Silver Spring, Maryland, United States of America; 4 Center for Infectious Disease Research, Walter Reed Army Institute of Research, Silver Spring, Maryland, United States of America; Duke University, UNITED STATES OF AMERICA

## Abstract

New HIV vaccine approaches are focused on eliciting broadly neutralizing antibodies. We characterized early gamma-delta (γδ) T cell responses starting from pre-acquisition and during acute HIV infection (AHI) in participants previously characterized for neutralization breadth development. We found significant differences in γδ T cell surface marker expression in participants that developed neutralization breadth compared to those that did not. Activation of γδ T cells occurred within the first weeks of HIV acquisition and associated with viral load. Expression of CD16 on Vδ1 T cells and CD57 on Vδ2 T cells were found to be significantly higher in broad neutralizers during AHI, and associated with the development of neutralization breadth years later. In addition, the levels of CD16 on Vδ1 T cells was associated with early production of founder virus Env-specific IgM. Thus, γδ T cells may promote development of neutralization breadth, which has implications for HIV vaccine strategies.

## Author summary

Vaccination against HIV-1 remains elusive due to the sequence diversity of the HIV-1 envelope. Several current HIV-1 vaccine designs are focusing on generation of broadly neutralizing antibodies (bNAbs). However, no HIV-1 vaccine has successfully elicited bNAbs. During natural infection, a minority of people living with HIV (PLWH) develop bNAbs. Therefore, there is great interest in studying the events that lead to bNAb generation in these study participants. γδ T cells express a limited repertoire of T cell receptor (TCR) V-regions and, in contrast to conventional CD4 and CD8 T cells, are not restricted by MHC-I or MHC-II. Here, we investigated γδ T cells prior to HIV-acquisition and during acute HIV infection (AHI) in individuals previously identified as ‘broad’ and ‘non-broad’ neutralizers, and determined their relationship with viral replication as well as bNAb elicitation. Expression levels of CD16 on Vδ1+ T cells as well as CD57 on Vδ2+ T cells during AHI were higher in participants that developed breadth and were significantly associated with the development of neutralization breadth years later. Our results suggest that γδ T cells might be important for early promotion of B cell engagement following HIV-1 acquisition and subsequent development of neutralization breadth.

## Introduction

Vaccination is considered a key tool to prevent acquisition and transmission of infectious pathogens. However, vaccination against HIV-1 remains elusive due to the sequence diversity of the HIV-1 envelope (glyco)protein (Env) and many other factors. Several current HIV-1 vaccine designs are focusing on generation of broadly neutralizing antibodies (bNAbs) [1–4]. However, no HIV-1 vaccine has successfully elicited bNAbs. During natural infection, a minority of people living with HIV (PLWH) develop bNAbs [5–7]. Therefore, there is great interest in studying the events that lead to bNAb generation in these study participants. Several viral [8–10] and immunological [11–14] factors have been reported to impact bNAb generation. However, most of these studies focused on the chronic phase of infection. Using the RV217 acute HIV infection (AHI) cohort [15], we have previously shown that B cell activation and engagement with founder Env within the first month of viremia are predictive of the development of neutralization breadth years later [16]. Understanding the innate and adaptive immune pathways that promote B cell activation in response to HIV-1 infection could inform HIV-1 vaccine design for the elicitation of neutralization breadth.

Gamma delta (γδ) T cells are important in the immune response to various types of pathogens ranging from viruses to bacteria and fungi [17]. γδ T cells express a limited repertoire of T cell receptor (TCR) V-regions and, in contrast to conventional CD4 and CD8 T cells, are not restricted by MHC-I or MHC-II. Furthermore, they share features with other innate like T cells, such as activation by cytokines in the absence of TCR signal [18] or by activating receptors usually associated with NK cell functions [19,20]. In humans, the most common subset in peripheral blood is defined by expression of the Vδ2 chain and recognize non-peptide phosphoantigens produced by some bacteria and protozoa such as *Plasmodium falciparum* [21,22]. These phosphoantigens are sensed by the butyrophilin-3A1 and butyrophilin-2A1 complex. Vδ1 T cells are abundant in mucosal tissues but are also found in peripheral blood, albeit at a lower frequency, and the antigens recognized by their TCR are less well defined [23,24]. One unique feature of γδ T cells is their capacity to become antigen presenting cells [25]. This includes following interaction with opsonized target cells by CD16, the low affinity FcγRIII, at the surface of γδ T cells [26,27]. γδ T cells can also promote humoral immune responses directly by stimulating the maturation of B cells [28] or indirectly by stimulating the differentiation of T follicular helper (Tfh) cells [29,30]. Thus, γδ T cells bridge innate and adaptive immune responses.

HIV-1 has a profound impact on γδ T cells. Early reports showed that Vδ1 T cells are expanded in PLWH [31,32]. In contrast, Vδ2 T cells were found to be decreased and poorly responsive to TCR stimulation in PLWH [33]. As a result of their susceptibility to HIV-1 infection, Vδ2 T cells have been found to contribute to the viral reservoirs present in PLWH on antiretroviral therapy (ART) [34]. Additionally, γδ T cells have been reported to have anti-HIV activity by producing β-chemokines that can block viral entry or by direct cytotoxicity against HIV-infected cells (reviewed in [35]). Thus, γδ T cells could impact HIV disease by modulating the adaptive immune system and by influencing viral replication either by their anti-viral activity or by supporting viral replication.

Here, we investigated γδ T cells prior to HIV-acquisition and during acute HIV infection (AHI) in RV217 individuals previously identified as ‘broad’ and ‘non-broad’ neutralizers, and determined their relationship with viral replication as well as bNAb elicitation. We found that Vδ2+ T cells were significantly reduced during early AHI and their loss was associated with viral load. On the contrary, Vδ1 T cells expansion occurred only during chronic HIV infection (CHI), and was not associated with viral load. Expression levels of CD16 on Vδ1+ T cells as well as CD57 on Vδ2+ T cells during AHI were higher in participants that developed breadth and were significantly associated with the development of neutralization breadth years later. Furthermore, levels of CD16 expression on Vδ1+ T cells were associated with early founder Env specific IgM production. Our results suggest that γδ T cells might be important for early promotion of B cell engagement following HIV-1 acquisition and subsequent development of neutralization breadth.

## Materials and methods

### Ethics statement

Samples from the previously described RV217 ECHO [15] cohort were utilized. Consenting adults from key populations in Kenya, Uganda, Tanzania, and Thailand were enrolled at clinical research sites. The protocol was approved by appropriate local review boards (Kenya Medical Research Institute Ethics Review Committee FWA00002066, National Institute for Medical Research FWA00002632, Mbeya Medical Research Ethics Committee FWA00032379, Uganda National Council for Science and Technology National HIV/AIDS Research Committee FWA00001293, and Royal Thai Army Medical Department IRB FWA 00001813) and the Walter Reed Army Institute of Research IRB (FWA 00000015), and written informed consent was obtained from all participants in their written languages.

### Study participants

Participants were screened twice weekly for HIV-1 RNA by finger pricks and nucleic acid amplification testing (NAAT; Aptima HIV-1 RNA Qualitative test, Hologic Inc., San Diego, CA). Participants with reactive NAAT (HIV RNA^+^) were enrolled in a second phase of the study that included intensive sampling of larger blood volumes throughout AHI and into CHI. Individuals were monitored longitudinally over time twice weekly for viral load and rising and declining viral loads allowed for identification of a peak and setpoint viral load timepoint [15]. All participants tested and determined to be living with HIV-1 were referred to care providers for management of the infection based on national guidelines, which did not incorporate routine treatment of acute infection until the START trial results were announced in 2015. From that point onward everyone was treated, regardless of national treatment initiation criteria. Prior to that time, we treated anyone with severe acute retroviral syndrome and did not prevent anyone from starting treatment if they and their physician chose to initiate treatment. Treatment was usually available at no cost through host nation care and treatment programs. The cases presented in this study include 22 selected RV217 participants described in a previous study that investigated the development of neutralization breadth [16]. Eleven of the participants were identified as broad neutralizers (defined as demonstrating neutralization breadth > 70% on a panel of 34 diverse pseudoviruses), and 11 were identified as non-broad neutralizers (defined as demonstrating < 35% neutralization breadth after 3 years of being in the study). None of these study participants had a super infection and five participants that developed neutralization breadth had confirmed multiple founder viruses [9]. Samples from these individuals were selected at four longitudinal time points as defined: prior to the detection of HIV-1 RNA (pre-acquisition), peak viral load (VL) (median days since first positive test for HIV-1 RNA = 18 days), set point VL (median days since first positive test for HIV-1 RNA = 42), and CHI (median days since first positive test for HIV-1 RNA = 980).

### Flow cytometry

Cryopreserved PBMC samples were thawed in media containing 20% fetal bovine serum (Millipore Sigma, Burlington, MA). Cell counts and viabilities were assessed using trypan blue and a countess II instrument (ThermoFisher, Waltham, MA). Cells were washed and stained with Aqua Live/Dead stain (Molecular Probes) for 30 minutes at room temperature (RT), washed again, and blocked using normal mouse IgG (ThermoFisher) 15 minutes at RT. Following washes, the cells were surface stained at RT for 30 minutes with CD69 BB660 (FW50, (BD Biosciences, San Jose, CA), ⍺4β7 AF-647 (Act-1, Invitrogen), CD8 APC-Cy7 (RPA-T8, BD Biosciences), HLA-DR BV480 (G46-6, BD Biosciences), CD19 BV570 (SJ25C1, BD Biosciences), CD14 BV570 (M5E2, BD Biosciences), CD3 BV605 (SK7, BD Biosciences), CCR7 BV650 (2-L1-A, BD Biosciences), TCRγδ BV711 (11F2, BD Biosciences), CD45RO BV786 (UCHL1, BD Biosciences), CD57 BUV395 (NK-1, BD Biosciences), CD16 BUV496 (3G8, BD Biosciences), TCR Vδ2 BUV563 (B6, BD Biosciences), CD56 BUV615-P (B159, BD Biosciences), PD-1 BUV661 (EH12.1, BD Biosciences), CD38 BUV737 (HB7, BD Biosciences), CD4 BUV805 (SK3, BD Biosciences), and TCR Vδ1 PE-Cy7 (TS8.2, ThermoFisher). The cells were then washed and sorted/analyzed on a ThermoFisher BigFoot Spectral Cell Sorter. The complete gating strategy is shown in Fig A in S1 File.

### Soluble marker measurement

Levels of 59 soluble biomarkers were measured from plasma samples via a combination of customized Luminex-based multiplex assays, and single-plex ELISAs per manufacturers’ protocols. Thirty-seven markers were measured via Bio-Plex Pro Human Inflammation Panel 1 (Bio-Rad, Hercules CA); twenty-one markers were assayed using Milliplex MAP Human High Sensitivity T Cell panel; and sCD14 was assayed by Human Quantikine ELISA (R&D Systems, Minneapolis MN). Luminex data were collected on a FlexMap 3D system (Luminex Corp, Austin TX) and ELISA data on a VersaMax microplate reader (Molecular Devices, Sunnyvale CA). All data were analyzed in Prism version 8.0 for Mac OS X (GraphPad, La Jolla CA) using a sigmoidal 4-parameter fit standard curve.

### RNA sequencing

Whole transcriptomics (coding and non-coding RNA) was performed by RNA-sequencing (RNA-seq) from sorted cells as described previously [36,37]. Briefly, 200 cells were directly sorted into 1x lysis buffer supplemented with 6U RNase inhibitor (both Takara Bio, Shiga, Japan). Post sort, sample tubes were vortexed, incubated at RT for 2 minutes, and snap frozen on dry ice prior to storage at −80˚ C. Upon thawing, cDNA synthesis was performed using the SMART-Seq v4 Ultra Low Input RNA Kit (Takara Bio) as per the manufacturer’s instructions. A total of 150–300 pg amplified cDNA was used as input for library preparation. Sequence-ready libraries were generated using Nextera XT DNA Library Prep Kit and quantitated on a MiSeq instrument using Reagent Kit v2 Nano (300 cycles, Illumina, San Diego, CA). All libraries were sequenced on the NovaSeq 6000 using S4 & S2 Reagent kits v1.5 (300 cycles) per manufacturer’s protocol (Illumina). Raw sequencing data were converted to FASTQ using *bcl2fastq2* v.2.20.0 (Illumina). The raw reads were aligned to the human reference genome (GRCh38) using STAR (v2.6.1d) [38] with default parameters, allowing for splicing junction mapping. Post-alignment, read counts were quantified using RSEM (v1.3.1) [39]. The count data were normalized and subjected to differential expression analysis using edgeR (v4.0.16) [40]. Genes with an adjusted p-value < 0.05 and a fold change > 2 were considered differentially expressed. DEGs’ fold changes and their statistical significance were visualized by volcano plots generated using the ggplot2 [41] package. Gene set enrichment analysis (GSEA; [42,43]) was performed as outlined by the Broad Institute and employing the Molecular Signatures Database (MSigDB) and the Kolmogorov-Smirnov test, utilizing a threshold for the normalized enrichment score (NES) to ensure relevance. UMAP (Uniform Manifold Approximation and Projection) analysis was performed using the R package UMAP (0.2.10.0 version), specifically targeting differentially expressed genes (DEGs) identified with a False Discovery Rate (FDR) threshold of 0.1. The RNA-seq data have been deposited in NCBI’s Gene Expression Omnibus and are accessible through GEO accession number GSE271442.

### Electro-chemiluminescence immunoassay (ECLIA)

The described multiplex ECLIA methodology is based on the Mesoscale U-PLEX platform utilizing 10-spot ECLIA plates (MSD, Gaithersburg, MD) and performed as previously described [44]. Briefly, biotinylated antigens were diluted to concentration of 300nM using coating diluent (1x PBS with 0.5% BSA) and linked with a unique U-plex linker provided by the U-PLEX platform (MSD), vortexed, and incubated at RT for 30 min. The U-PLEX-coupled antigen solutions were brought up to 6 ml with Stop Solution, creating a 1x multiplex coating solution. Plates were coated with the cocktail of antigens and incubated at RT for 1h on a Titramax plate shaker (Heidolph, Schwabach, Germany), shaking at 700 rpm. After incubation, the plates were washed with a working solution of 1x MSD Wash Buffer (MSD) three times. Sera were diluted 1:500 with Diluent 2 (MSD), added to each well and incubated at RT for 1h on a plate shaker. Plates were washed three times with 1x MSD Wash Buffer and incubated with detection antibody, SULFO-TAG goat anti-human antibody (diluted to 1 µg/ml in Diluent 3 (MSD)). Plates were sealed and incubated at RT for 1h on a plate shaker (700 rpm). After washing, MSD Read Buffer T was added and the plates read on the MESO QuickPlex SQ 120 (MSD), per manufacturer’s instructions. Recombinant *P. falciparum* (3D7 strain) proteins (PfMSP-1p42, PvMSP-1, PfAMA-1, Rifin, Pfs25, Pfs16, CelTOS, GTP-binding protein (GBP), ETRAMP4, ETRAMP5) were produced at Genscript (Piscataway, NJ). Peptides derived from the *Anopheles gambiae* salivary gland protein (gSG6; peptides 1 and 2 [45], circumsporozoite protein (CSP; representing the major repeat PfCSP NANP), PvCSP Repeat 1, Pv N-terminus, the PfCSP and Pv CSP C-termini [44] were synthesized by Atlantic Peptides).

### Quantification and statistical analysis

Prism v.8.0.2 (GraphPad Software, Inc., San Diego, CA) and R (3.6.3) through R Studio (1.1.453, R Consortium, Boston, MA) were employed for all statistical analyses and graphical representations. Differences in the distribution of categorical variables were tested using a chi-square test. Longitudinal analysis of Vδ1+ and Vδ2+ T cell frequency, ratio, and phenotype was conducted using a mixed-effects ANOVA test with Šídák’s multiple comparisons test. Correlation values were determined utilizing Spearman’s rank-correlation analysis. Comparisons between broad and non-broad neutralizers pre- and post-HIV acquisition were conducted using a two-way mixed-effects analysis with Tukey’s multiple comparisons test. Logistic regression was used to estimate the quantitative relation between levels of Vδ1+ and Vδ2+ T cell markers measured at each timepoint. Vδ1+ and Vδ2+ T cell marker levels were categorized into ordinal groups based on quantile distribution (above or below 33%, 50%, or 66%), and logistic regression analysis was employed to investigate the association between these categorized levels and neutralization breadth. Forest plots were used to visualize the results obtained from logistic regression. The performance of prediction on status of broad neutralization based on each marker was evaluated through receiver operating characteristic (ROC) curve and area under the curve (AUC) value of the ROC curve. Cumulative incidence curves were used to illustrate the likelihood of becoming broad neutralizers over time for group of subjects with levels of T cell markers passing thresholds of quantiles. Significance evidence was determined using a Cox proportional hazards model via Likelihood ratio test (LRT) at significant level of 5%. Confidence intervals at 95% confidence level are shown as shaded areas around each curve.

## Results

### Vδ1+ and Vδ2+ T cells are activated during AHI

**Fig 1:**
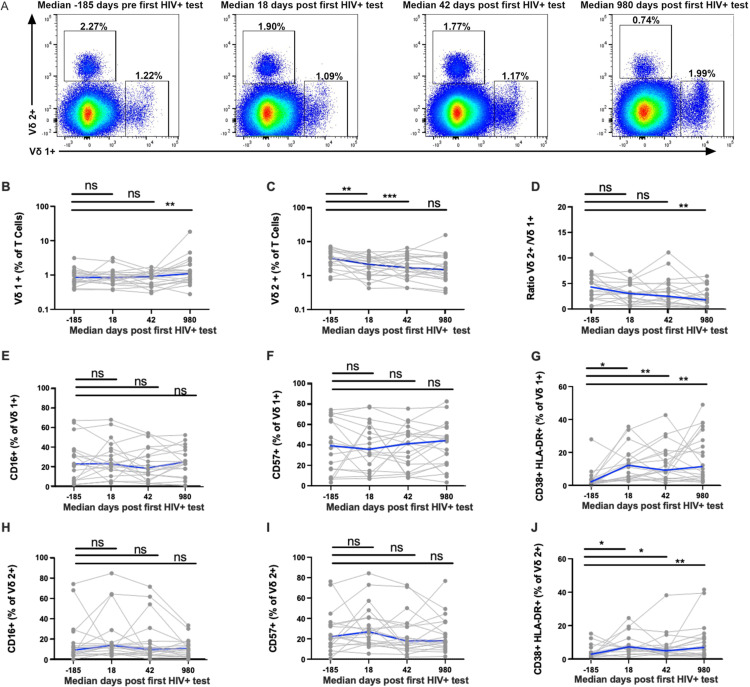
Vδ1+ and Vδ2+ T cells are activated during AHI. (A) Dynamics of Vδ1+ and Vδ2 T cell activation during AHI. Representative flow plots of Vδ1+ and Vδ2+ T cell frequencies at median −185 prior to HIV+ test (pre-infection) as well as at peak viral load (median: 18 days), setpoint viral load (median 42 days), and chronic infection (median: 980 days) following the first detectable HIV RNA (HIV+). (B–D) Longitudinal analysis of Vδ1+ and Vδ2+ T cell frequency and ratio. (E–J) The expression of effector molecules (CD16 and CD57), and activation markers (CD38+ HLA-DR+) on Vδ1+ and Vδ2+ T cells. Each participant is indicated by a grey line and the blue line indicates the median. *P < 0.05, **P < 0.01, ***P < 0.001.

First, to determine the dynamics of γδ T cell responses during AHI and CHI, Vδ1+ and Vδ2+ T cells from 22 selected RV217 participants, who were previously identified as ‘broad’ (N = 11) and ‘non-broad’ neutralizers (N = 11) [16] (Table 1) were sorted (Fig 1A and S1) from PBMC samples collected at median −185 (pre-acquisition), 18 (corresponding to peak VL), 42 (corresponding to set point VL), and 980 days (CHI) following the first detection of HIV RNA in the blood. Longitudinal changes in γδ T cell frequency and expression markers were assessed. Vδ1+ T cells significantly increased by a median of 1.1-fold and up to 2.2-fold during CHI compared to pre-acquisition (Fig 1B). On the contrary, a significant decrease in Vδ2+ T cell frequency was observed at 18 and 42 days following the first HIV+ test by a median decrease of 1.35-fold for both time points and up to 2.2 and 2.0-fold respectively (Fig 1C). As a result, the ratio of Vδ2+ to Vδ1+ T cell frequency was significantly lower during CHI compared to pre-acquisition (Fig 1D). While no significant differences were observed in the expression of CD16+ or CD57+ by Vδ1+ T cells or Vδ2+ T cells at any time point (Fig 1E, F , H, I), a significant increase in Vδ1+ and Vδ2+ T cells co-expressing the activation markers CD38 and HLA-DR was observed at all time points post HIV-acquisition (Fig 1G, J). Additionally, CD45RO and α4β7 were included in our study as important markers of memory status and gut homing potential that could be modulated by HIV. We found a modest decrease in Vδ2+ T cells and increase in Vδ1+ T cells expressing CD45RO at days 18 and 42 respectively (panels A and C of Fig B in S1 File). There was no change in α4β7 levels by Vδ1+ T cells or Vδ2+ T cells at any time point (panels B and D of Fig B in S1 File). Our results demonstrate that the frequency of Vδ2+ T cells decrease early during AHI, but Vδ1+ T cells expand during CHI.

**Table 1:** Demographics of study participants.

	Broad neutralizers	Non-broad neutralizers	P value
Total number of participants	11	11	
Gender*			0.51
Male	2	3	
Female	8	6	
Transgender	1	2	
Country*			0.05
Thailand	3	5	
Uganda	2	0	
Kenya	1	5	
Tanzania	5	1	
Multiple founders*			0.01
Yes	5	0	
No	6	11	
Days since 1^st^ HIV+ Test^#^			
Pre-acquisition	N = 7; −158 (−12;−377)	N = 11; −334 (−11;−700)	0.17
Day 18	N = 10; 17 (0–25)	N = 10; 18 (1314151617181920–21)	0.96
Day 42	N = 11; 45 (31–99)	N = 9; 40 (26–48)	0.84
Day 980	N = 9; 1101 (681–1672)	N = 11; 992 (709–1517)	0.66
Peak breadth^$^	80.8 (70.6–94.1)	24.6 (11.8–32.4)	<0.0001
Potency^$^	94.8 (70.0–114.0)	36.3 (28.0–48.0)	<0.0001
Infecting subtype*			0.54
A1	5	3	
A1/C	0	1	
A1/C/D	1	0	
C	1	1	
D	0	1	
D/C	1	0	
CRF01_AE	3	5	

* Difference in distribution was tested using a chi square test.

# Data is shown has N; median (range).

$ Data is shown has median (range).

Repeated malaria exposure is known to impact the frequency and phenotype of γδ T cells [46,47], including increasing the expression levels of CD16 [48] and CD57 [49]. Therefore, we measured levels of antibodies related to exposure to mosquito bites and malaria antigens in the participants included in this study pre-HIV acquisition and tested for correlations with the frequency and phenotype of γδ T cells (Fig C in S1 File). A constant pattern of positive correlations was found with the frequency of Vδ2+ T cells and the antibody levels against several antigens associated with exposure to mosquito bites and malaria. Similar results were found for CD8 expression by Vδ1+ T cells. We also observed positive associations between the antibody levels against *P. falciparum* Sporozoite antigen CSP C-terminal peptide (PfCSP c-term) and expression of CD16 by Vδ1+ T cells as well as CD45RO by Vδ2+ T cells. This suggests that some of the variability in the frequency and phenotype of γδ T cells pre-HIV acquisition might come from exposure to malaria.

### Viral load is associated with Vδ1+ and Vδ2+ T cell activation

**Fig 2:**
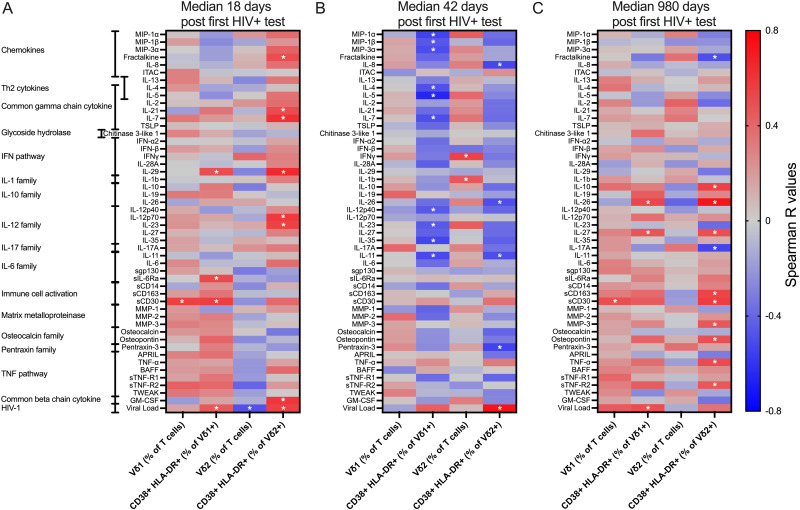
Viral load is associated with Vδ1+ and Vδ2+ T Cell activation. Association between soluble marker levels and Vδ1+ and Vδ2+ T cell frequency and activation. The heat map shows Spearman R values for associations between soluble markers, Vδ1+ and Vδ2+ T cell frequency, and Vδ1+ and Vδ2+ T cell activation at median 18 (A), 42 (B), and 980 (C) days post first HIV+ test. *P < 0.05.

Next, to investigate what factors associate with Vδ1+ and Vδ2+ T cell activation, we measured the plasma levels of several inflammatory markers. Plasma levels of B cell activation factor (BAFF), sCD30, sCD14, and TNFα were found to be elevated at all time points whereas IL-1β, IL-4, IL-6, IL-8, and IL-13 were elevated only during AHI (Fig D in S1 File). Several soluble factors showed positive associations with Vδ2+ T cell activation during early AHI, but those associations were not maintained at other time points (Fig 2A). sCD30 was the only measured parameter that associated with the increased frequency of Vδ1+ T cells during CHI (Fig 2C). However, adding CD30 to PBMC in vitro did not activate Vδ1+ T cells (Fig E in S1 File). Since sCD30 is released by activated T cells [50,51], we investigated if conventional CD4 or CD8 T cell activation was associated with Vδ1+ T cells frequency. Only CD4 T cell activation during CHI was associated with Vδ1+ T cell frequency at the concurrent time point (Fig F in S1 File). Of note, markers of monocyte activation commonly used as proxies for microbial translocation, sCD14 and sCD163, were not associated with either the frequency nor activation of Vδ1+ and Vδ2+ T cell at any time post HIV-acquisition (Fig 2A–C). VL was also included in the analysis as an important factor that could contribute to the change in frequency and activation of Vδ1+ and Vδ2+ T cells. In fact, VL was consistently associated with activation of Vδ1+ T cells (Fig 2A–C) and was also associated with the decreased frequency and increased activation of Vδ2+ T cells during early AHI (Fig 2A, B), suggesting that VL may be driving Vδ1+ and Vδ2+ T cell activation.

### IFN-responsive and anti-viral pathways are upregulated in γδ T cells during AHI

**Fig 3:**
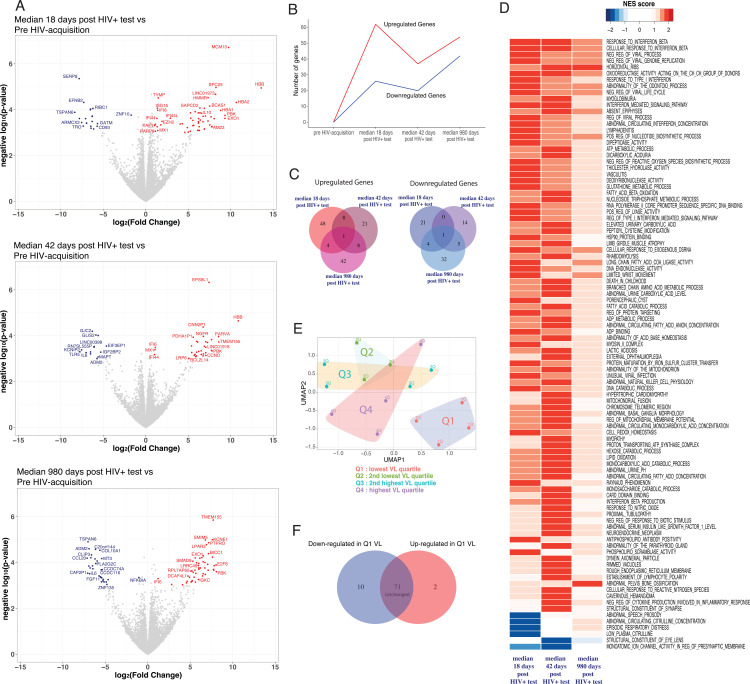
IFN-responsive and anti-viral pathways are induced in γδ T cells post-HIV-1 acquisition. (A) RNA-Seq was performed on sorted total γδ T cells obtained from the longitudinal PBMC samples at pre and post-HIV-1 acquisition time points. Volcano plots depict upregulated (red) or downregulated (blue) genes compared to pre-acquisition at three post-acquisition time points. Highlighted genes have a -log_10_(p-value) ≥ 3 and a log_2_(Fold Change) of 1 or −1 (corresponding to p ≤ 0.001, and fold change of 2 or 1/2, in a generalized linear model). (B) The temporal dynamics of the upregulated and downregulated genes are shown longitudinally post-HIV-1 acquisition. (C) Shared and unshared differently expressed genes compared to pre-acquisition between all three post-acquisition time points are highlighted as a Venn diagram. Increased genes are shown in shades of red and decreased genes are shown in shades of blue. (D) Gene expression patterns were subjected to Gene Set Enrichment Analysis (GSEA), and upregulated and downregulated pathways in post-acquisition time points compared to pre-acquisition are displayed as a Normalized Enrichment Score (NES) heat map. (E) UMAP analysis of the DEGs (FDR below 0.1) at median 18 days post first HIV-1+ test. Study participants were grouped in quartiles based on their set point VL. Q1 (red dots) and Q4 (purple dots) have the lowest and highest set point VL respectively. (F) Venn diagram showing the number of down or up-regulated genes (p value < 0.05) in Q1 set point VL compared to Q2–Q4 participants.

To gain further insight to the γδ T cell response following HIV-1 acquisition, sorted total γδ T cells were processed for bulk RNA sequencing and gene expression post HIV-acquisition was compared to pre-acquisition (Fig 3A and S2 File). The highest number of genes with increased expression was found at peak VL (median 18 days from first detectable HIV+ test; Fig 3B). The number of differentially expressed genes (DEGs) decreased at setpoint viral load (day 42 post first HIV+ test) and increased in CHI (Fig 3B). Little overlap in DEGs was observed between any of the time points tested (Fig 3C). We then performed GSEA to determine if pathways were modulated following HIV-1 acquisition (Fig 3D). Several pathways related to IFN- and anti-viral responses were upregulated during AHI with most returning to pre-acquisition levels during CHI. We then investigated if DEGs in γδ T cells could impact setpoint VL. Levels of genes with a q < 0.1 at day 18 post first HIV+ test were used to generate a UMAP and study participants were grouped based on setpoint VL quartiles (Fig 3E). Participants with the lowest setpoint VL (Q1) clustered away from all other study participants (Q2–Q4), suggesting a different gene expression profile in participants with the lowest set point VL. We then analysed each DEG individually and found that this separation was attributed to lower levels of DEG in participants with the lowest set point VL (Fig 3F and S2 File). Thus, our data suggest that γδ T cell induction of IFN-responsive and anti-viral pathways during AHI is driven by viral replication and may not directly contribute to viral control.

### Phenotypic differences in Vδ1+ and Vδ2+ T cells between participants that developed or failed to develop neutralization breadth

**Fig 4:**
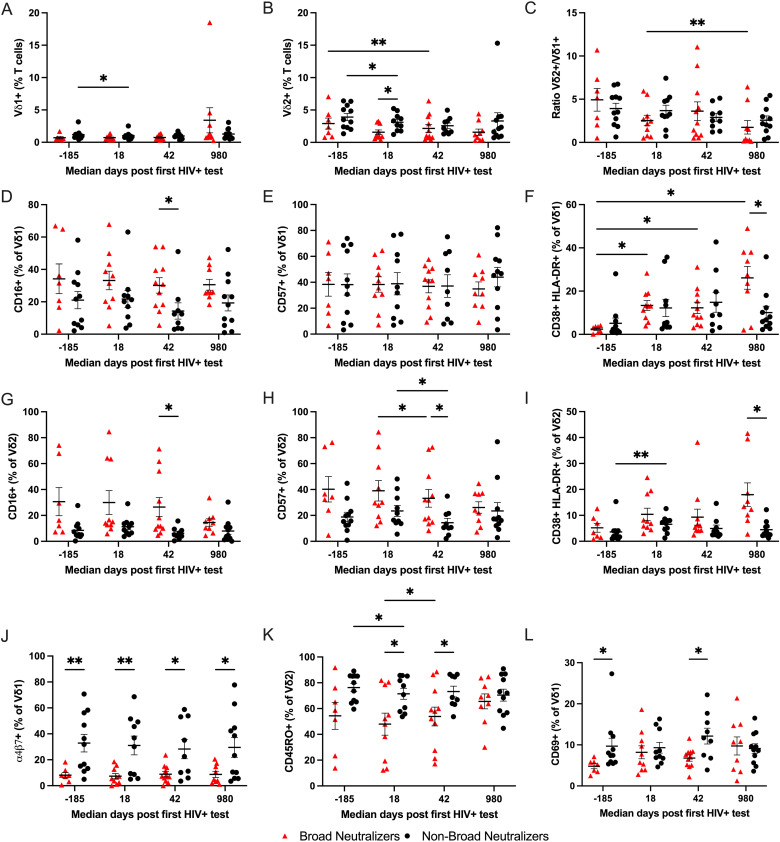
Differences in phenotype of Vδ1+ and Vδ2+ T cells between broad and non-broad neutralizers. Comparison between the frequency and phenotype of Vδ1+ and Vδ2+ T cells between broad and non-broad neutralizers pre- and post-HIV acquisition. Each data set is presented as the mean ± SEM. Broad and non-broad neutralizers were compared using a two-way mixed-effects ANOVA test. N = 7 and N = 11 for broad and non-broad neutralizers respectively pre-HIV acquisition, N = 10 for broad and non-broad neutralizers at day 18 post first HIV+ test, N = 11 and N = 9 for broad and non-broad neutralizers respectively at day 42 post first HIV+ test, N = 9 and N = 11 for broad and non-broad neutralizers respectively at day 980 post first HIV+ test. *P < 0.05 **P < 0.01.

The RV217 study participants included in this study were previously characterized for the development, or lack of, neutralization breadth [16]. Participants that developed neutralization breadth had a higher peak VL, but no differences were observed in set point VL or CHI VL (Fig G in S1 File). We then compared the frequencies and phenotypes of Vδ1+ and Vδ2+ T cells between participants who developed neutralization breadth or failed to develop neutralization breadth as well as changes over time within each group using a two-way mixed-effects ANOVA test. The frequency of Vδ2+ T cells was lower in broad neutralizers at peak VL (median 18 days post first HIV+ test; Fig 4B), but no significant difference in the frequency of Vδ1+T cells (Fig 4A) nor the Vδ2+ to Vδ1+ ratio (Fig 4C) between broad and non-broad neutralizers was found at any time point. There was an increase in CD16+ expressing Vδ1+ T and Vδ2+ T cells in participants that developed neutralization breadth at setpoint VL (median 42-days post first HIV+ test; Fig 4D, G) but we found no differences in the levels of CD57 by Vδ1+ T cells (Fig 4E). On the contrary, Vδ2+ T cells exhibited higher levels of CD57 in participants who developed neutralization breath at setpoint VL (median 42-days post first HIV+ test; Fig 4H). We also found higher CD38 and HLA-DR co-expression by both Vδ1+ T and Vδ2+ T cells during CHI in participants who developed neutralization breath (Fig 4F, I). Co-expression of CD38 and HLA-DR increased over time in both groups, but it reached statistical significance only for participants who developed neutralization breath for Vδ1+ T cells and for participants that failed to develop breadth for Vδ2+ T cells. We observed a pattern of reduced expression of α4β7 and CD69 on Vδ1+ T cells as well as of CD45RO on Vδ2+ T cells in participants who developed neutralization breath at several time points (Fig 4J–L). Our results suggest that participants who developed neutralization breadth have a differential phenotype of Vδ1+ and Vδ2+ T cells during AHI and, for some markers, prior to HIV acquisition. As a result of this observation, we then compared expression of CD16, CD57, CD69, α4β7, and co-expression of CD38 and HLA-DR on conventional CD4 and CD8 T cells as well as NK cells between participants who developed neutralization breadth and those who did not. While the levels of some of these markers changed over time following HIV acquisition, the only significant difference between participants who developed neutralization breadth and those who did not was the co-expression of CD38 and HLA-DR at the chronic time point for conventional CD4 and CD8 T cells (Fig H in S1 File). Thus, this indicates that the observed differences in the levels of CD16, CD57, CD69, and α4β7 are unique to γδ T cells.

Next, we compared γδ T cell gene expression between participants who developed neutralization breadth and those who did not (Fig I in S1 File and S3 File). Few genes remained significantly differentially expressed after correcting for multiple testing, with practically no overlap between time points. 18 genes at peak VL (median 18 days) were significantly increased in participants that developed neutralization breadth and the number of these genes declined over time. In contrast, the number of genes that significantly decreased expression in participants that developed neutralization breadth kept increasing over time. In accordance with our flow cytometry results, gene expression of CD16 (FcγR3A) and CD57 (B3GAT1) was higher in participants that developed neutralization breadth; however, these differences did not remain significant after correcting for multiple testing (S3 File). Overall, these results suggest that participants that developed breadth had a different Vδ1+ and Vδ2+ T cell profile.

### Expression of CD16 by Vδ1+ and CD57 Vδ2+ T cells during AHI is associated with the development of neutralization breadth

**Fig 5:**
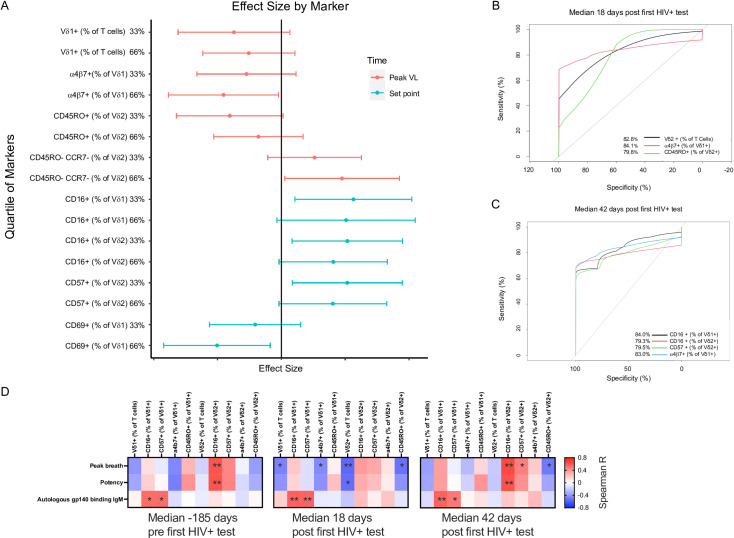
Vδ1 and Vδ2 T cell markers are associated with the probability of developing neutralization breadth. Odds ratios (OR, solid symbols) and 95% CI (error bars) are indicated on the forest plot (A) with a vertical line to indicate an OR of 1. Markers exceeding quantile thresholds with confidence interval excluding OR of 1 are significant. AUROC (B) indicate the neutralization breadth prediction accuracy for Vδ2+ T cells (82.8%), α4β7+ Vδ1 T cells (84%), and CD45RO+ Vδ2 T cells (79.8%) at peak VL. AUROC (C) indicate neutralization breadth prediction accuracy for the frequency of CD16+ Vδ1 T cells (84%), CD16+ Vδ2 T cells (79.3%), CD57+ Vδ2 T cells (79.3%), and α4β7+ Vδ1 T cells (83%) at set point viral load. (D) The heat map shows Spearman R values for associations between peak breadth, potency, autologous gp140 specific IgM at month one and the frequency and phenotype of Vδ1 and Vδ2 T cell pre-acquisition, median day 18, and median day 42 post first HIV+ test. *P < 0.05 **P < 0.01.

To explore the relationship between Vδ1+ and Vδ2+ T cells and neutralization breadth, Vδ1+ and Vδ2+ T cell marker levels were categorized into ordinal groups (above or below 33%, 50%, or 66% quantile) and logistic regression was performed controlling for VL and visualized as forest plots (Fig 5A). We found that participants who had high levels of CD16 expression (above 33% quantile) by Vδ1+ T cells at setpoint VL (median 42 days post first HIV+ test) were significantly more likely to develop neutralization breadth. High expression of CD69 (above 66% quantile) at setpoint VL (median 42 days HIV+) or of α4β7 (above 66% quantile) at peak VL (median 18 days HIV+) by Vδ1+ T cells were associated with lower odds of developing neutralization breadth. Participants with Vδ2+ T cells expressing high levels of CD57 (33% quantile) or CD16 (33% quantile) at setpoint VL (median 42 days HIV+) or double negative for CD45RO and CCR7 (66% quantile) at peak VL (median 18 days HIV+) were also significantly more likely to develop neutralization breadth (Fig 5A). Next, to maintain each parameter as a continuous variable, ROC analysis was carried out to evaluate the performance achieved by each marker in determining the status of broad neutralization. At peak VL (median day 18 HIV+), frequencies of Vδ2+ T cells, α4β7+ Vδ1+ T cells, and CD45RO+ Vδ2+ T cells had an AUC of 0.828, 0.841, and 0.798 respectively (Fig 5B). Similarly, at setpoint VL (median 42 days HIV+), CD16+ Vδ1+ T cells, CD16+ Vδ2+ T cells, CD57+ Vδ2+ T cells, and α4β7+ Vδ1+ T cells had an AUC of 0.84, 0.793, 0.795, and 0.83 respectively (Fig 5C). Overall, this suggests that expression of these markers by Vδ1+ and Vδ2+ T cells during AHI discriminate between participants that will develop neutralization breadth. In addition, we performed cumulative incidence curves. Participants with Vδ1+ T cells expressing CD16 above the median levels or with Vδ2+ T cells expressing CD57 above the first tertile at setpoint VL (42 days HIV+) were significantly more likely to develop neutralization breadth (Fig J in S1 File). No other marker could separate participants that developed neutralization breadth using cumulative incidence curves. Taken together, our results indicate that, during AHI, expression of CD16 and CD57 by Vδ1+ and Vδ2+T cells respectively is associated with the development of neutralization breadth during CHI.

Finally, we tested for associations between the phenotype of γδ T cells and peak breadth, potency, as well as autologous gp140-specific IgM at month 1 post HIV-acquisition, that we previously reported to be associated with development of neutralization breadth in the RV217 participants analyzed in this study [16]. Strikingly, we found that expression levels of CD16 and CD57 by Vδ1+ T cells pre- and post-HIV acquisition were associated with the plasma levels of founder gp140 specific IgM at month 1 post first HIV+ test (Fig 5D). Furthermore, levels of CD16 by Vδ2+ T cells pre-acquisition and at day 42 post first HIV+ test, corresponding to set point VL, correlated with breadth and potency. The phenotype of γδ T cells did not associate with the previously reported B cell phenotype during AHI [16] (Fig K in S1 File). These results suggest that CD16 expression by Vδ1+ T cells could promote early engagement of Env specific B cells during AHI.

## Discussion

Previous cross-sectional studies in PLWH suggested a role for γδ T cells in disease progression. Furthermore, γδ T cells are known to have the capacity to regulate B cell responses. In this study, we evaluated the frequency and phenotype of γδ T cells longitudinally starting from pre-HIV acquisition and during untreated AHI in participants with or without neutralization breadth [16], allowing us to evaluate the contribution of γδ T cells to initial control of viral replication and to the promotion of humoral immune responses. Vδ2+ T cells were depleted from peripheral blood as early as day 18 post first HIV-1 RNA positive test and their frequency was inversely associated with VL, suggesting a direct impact of HIV-1 on these cells. The reduced frequency of Vδ2 T cells in PLWH has been suggested to results from both direct infection [52] and indirect mechanism involving CCR5 and α4β7 signaling [53]. On the contrary, expansion of Vδ1+ T cells was observed only during CHI and was not associated with VL but with sCD30, a marker of T cell activation. Accordingly, Vδ1+ T cell frequency during CHI was associated with conventional CD4, but not CD8, T activation. Taken together, this suggests that similar factors might be driving the expansion of Vδ1+ T cells and CD4 T cell activation during untreated CHI. Microbial translocation has been associated with immune activation during CHI [54] and was suggested to be responsible for the increased frequency of Vδ1+ T cells in SIV infection [55]. However, we did not find an association between sCD14 or sCD163, two markers of monocyte activation used as surrogate for microbial translocation, and Vδ1 T cells frequency during CHI. HCMV has also been reported to contribute to chronic T cell activation in PLWH [56] and to drive the expansion of Vδ1+ T cells in transplant recipients following reactivation [57,58] and in primary immunodeficiency [59]. Thus, it is possible that HCMV could be one factor contributing to the expansion of Vδ1 T cells during CHI. A similar dynamic of early Vδ2+ T cells depletion preceding Vδ1+ T cells expansion has also been reported in SIV infection [60].

Furthermore, we found that both Vδ1+ and Vδ2+ T cells were activated during AHI, and this was directly associated with VL. The pattern of early depletion and activation of Vδ2+ T cells during AHI mirrors what we have previously reported for iNKT cells with the difference that iNKT cell activation was not associated with VL [61]. Changes in γδ T cell gene expression were more profound around the time of peak VL and were characterized by induction of several interferon or anti-viral related pathways. However, a strong induction of these pathways was not associated with lower viral replication. In fact, participants with a lower set point VL had a smaller change in γδ T cells gene expression, suggesting that change in γδ T cells gene expression is a consequence of HIV-1 replication. This is in accordance with our flow cytometry results showing that co-expression of the activation markers CD38 and HLA-DR during AHI was associated with viral load. Due to sample limitations, γδ T cell functions were not evaluated. Additional studies are needed to investigate if γδ T cell functions have an impact on HIV-1 progression. Overall, we found no clear evidence that peripheral γδ T cells could limit viral replication during AHI. However, the results from the gene expression analysis should be analyzed with caution as we sorted bulk γδ T cells and not Vδ1+ and Vδ2+ T cells individually.

However, we could identify several differences in the phenotype of circulating γδ T cells between participants that developed neutralization breadth and those that did not. The frequency of Vδ2+ T cells was lower at day 18 post first HIV-1 positive test in the participants that developed neutralization breadth, probably reflecting the higher peak VL in those participants. CD16 levels on both Vδ1+ and Vδ2+ T cells as well as CD57 levels on Vδ2+ T cells were found to be higher on participants that developed neutralization breadth. These differences were specific to γδ T cells as we did not observe differences in CD16 nor CD57 levels at any time points on conventional CD4 and CD8 T cells or NK cells between participants who developed neutralization breadth and those who did not. Due to the small number of participants included in our study, it will be important to confirm these findings in larger cohorts. However, the prospective cohort allowed us to show that expression of these two markers was not modulated by HIV-1 and that significant differences were already present pre-HIV acquisition for CD16 and CD57 levels on Vδ2+ T cells. Infections with HCMV and malaria have been reported to increase expression of these markers by γδ T cells [48,62]. In our cohort, the levels of antibodies against PfCSP c-term, indicative of exposure to malaria sporozoite, showed a positive association with CD16 expression on Vδ1+ T cells, suggesting that malaria exposure might contribute to the phenotype of γδ T cells associated with the development of neutralization breadth, but other unidentified factors might also be important. The distribution of expression level of the markers identified in this study and the associated quantiles were based on a subset of participants that met pre-specified criteria and may differ from that of the general population. This potential limitation is inherent in studies based on selected subsets, but we believe that our results provide valuable biological insights.

The mechanism by which γδ T cells could promote the development of neutralization breadth remains to be investigated. CD16 expression by Vδ1+ T cells was associated with B cell engagement during AHI, suggesting that these cells might promote the initial founder-specific naïve B cell response to HIV-1. One possibility is that γδ T cells could differentiate into antigen presenting cells following engagement of CD16 with opsonized target cells [26]. Since neither CD16 nor CD57 expression by Vδ2+ T cells was associated with B cell engagement during AHI, it is possible that these cells have a different mechanism of action compared to Vδ1+ T cells. Vδ2+ T cells may be involved in B cell differentiation pathways after the initial interaction between the founder Env and naive B cells. In support of this hypothesis, treatment with phosphoantigens and IL-2 to activate and expand Vδ2+ T cells during chronic SIV infection increased titers of Env-specific antibodies [63]. Additional studies could benefit from incorporating tissue sample, such as lymph nodes, analysis for γδ T cell responses when evaluating factors that influence the development of neutralization breadth. Understanding the mechanism by which γδ T cells promote the development of broadly neutralizing antibodies could help develop strategies targeting these cells to improve vaccine induced humoral immune responses, especially the induction of broad and potent neutralizing antibody to HIV-1 Env.

In conclusion, we have shown that loss of Vδ2+ T cell occurs early during AHI and is associated with VL while expansion of Vδ1+ T cells occurs later during CHI and is associated with CD4 T cell activation. Importantly, we observed higher levels of CD16 and CD57 on γδ T cells in participants that developed neutralization breadth. Levels of CD16 on Vδ1+ T cells were associated with early founder Env specific IgM levels, suggesting that CD16+ Vδ1+ T cells may promote B cell engagement with Env during AHI.

## Supporting information

S1 File Supplementary Figs A–K. (DOCX)

S2 File Differential gene expression comparing median days 18, 42, and 980 to pre-acquisition as well as comparing gene expression at day 18 between participants with low set point viral load (Q1) to the others. (XLSX)

S3 File Differential gene expression comparing participants with neutralization breadth to those without at four time points: pre-acquisition, median days 18, 42, and 980. (XLSX)

S4 File Minimal Data Set. (XLSX)
