# Supplementary material for: CD16 and CD57 expressing gamma delta T cells in acute HIV-1 infection are associated with the development of neutralization breadth
Source: PLoS Pathog. 2025 Jan 31;21(1):e1012916. doi: 10.1371/journal.ppat.1012916 (PMC11805418; doi:10.1371/journal.ppat.1012916)
Supplement: S1 File — (DOCX) [file ppat.1012916.s001.docx]

**
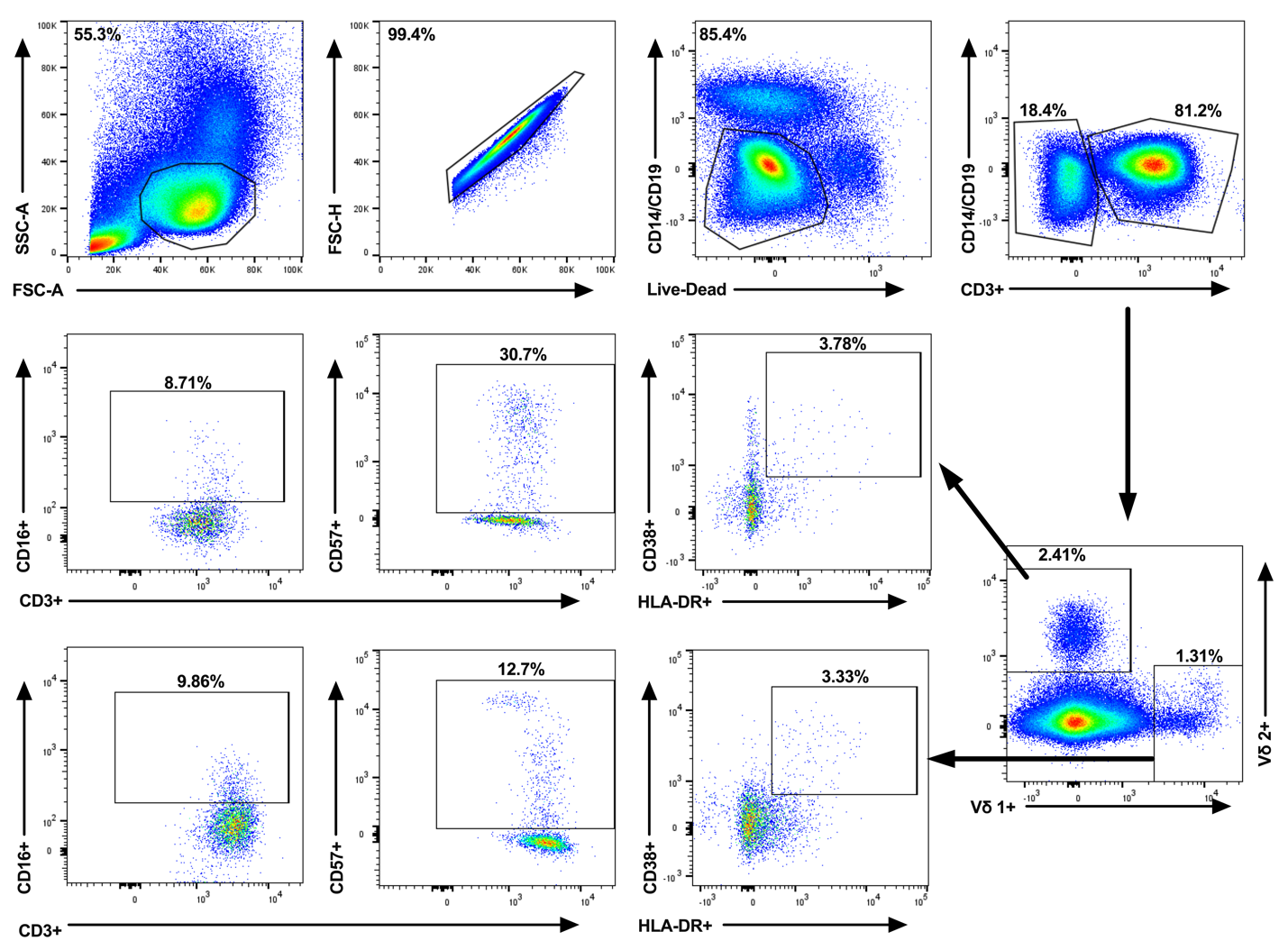
**

**Fig A.** Representative Vδ1+ and Vδ2+ T cell Gating Strategy. Representative gating strategy of Vδ1 and Vδ2+ T cell populations and levels of effector molecules (CD16 and CD57) and activation markers (CD38+ HLA-DR+).


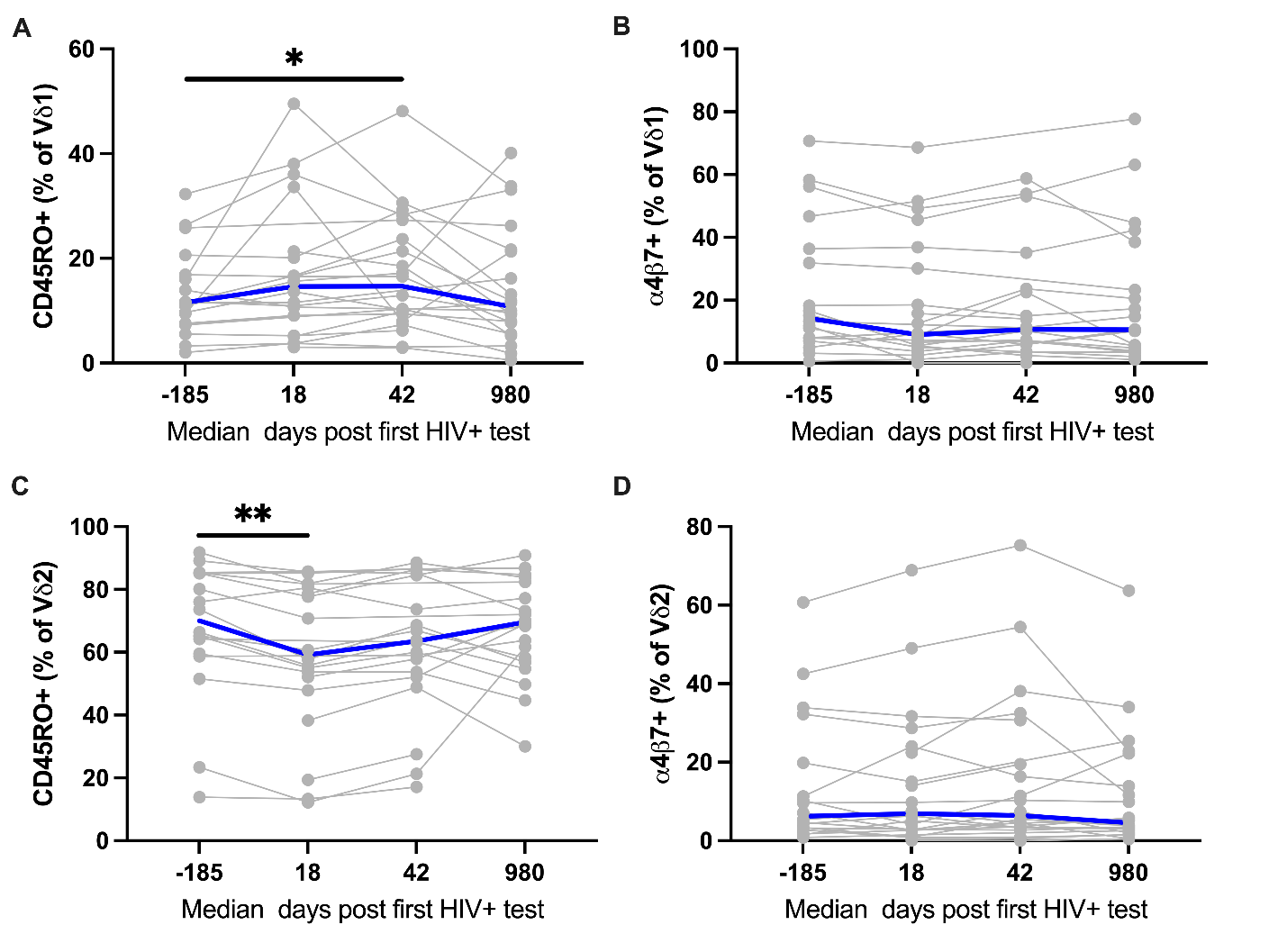


**Fig B.** The expression of CD45RO (A and C), and α4β7 (B and D) on Vδ1+ and Vδ2+ T cells. Each participant is indicated by a grey line and the blue line indicates the median. *P < 0.05, and **P < 0.01.

**
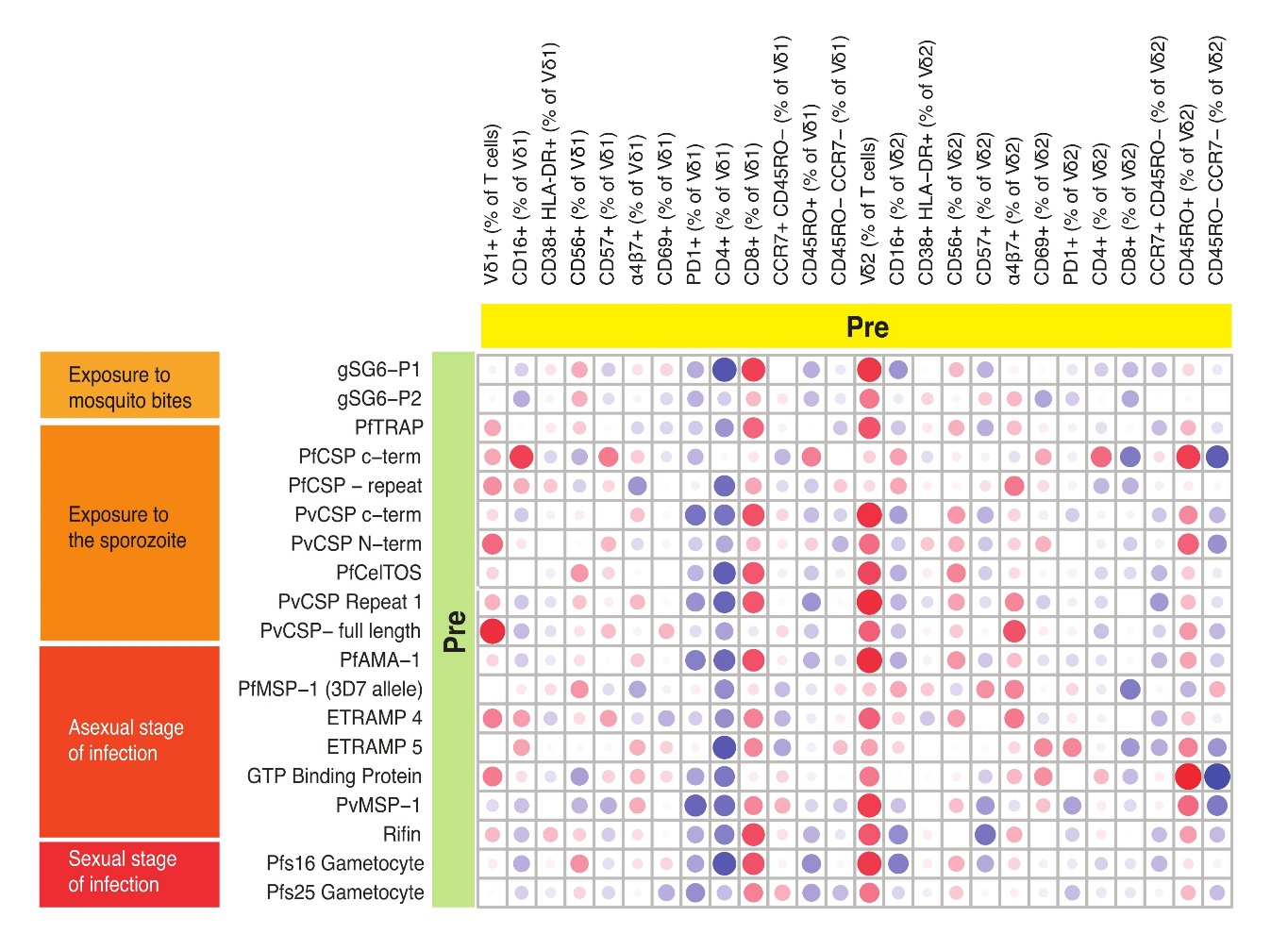
**

**Fig C.** Associations between Malaria exposure and γδ T cell frequency and phenotype pre- HIV-1 acquisition. Heat map showing Spearman Rho values between the frequency and phenotype of γδ T cell pre-HIV acquisition with antibodies level indicative of malaria exposure pre-HIV acquisition.


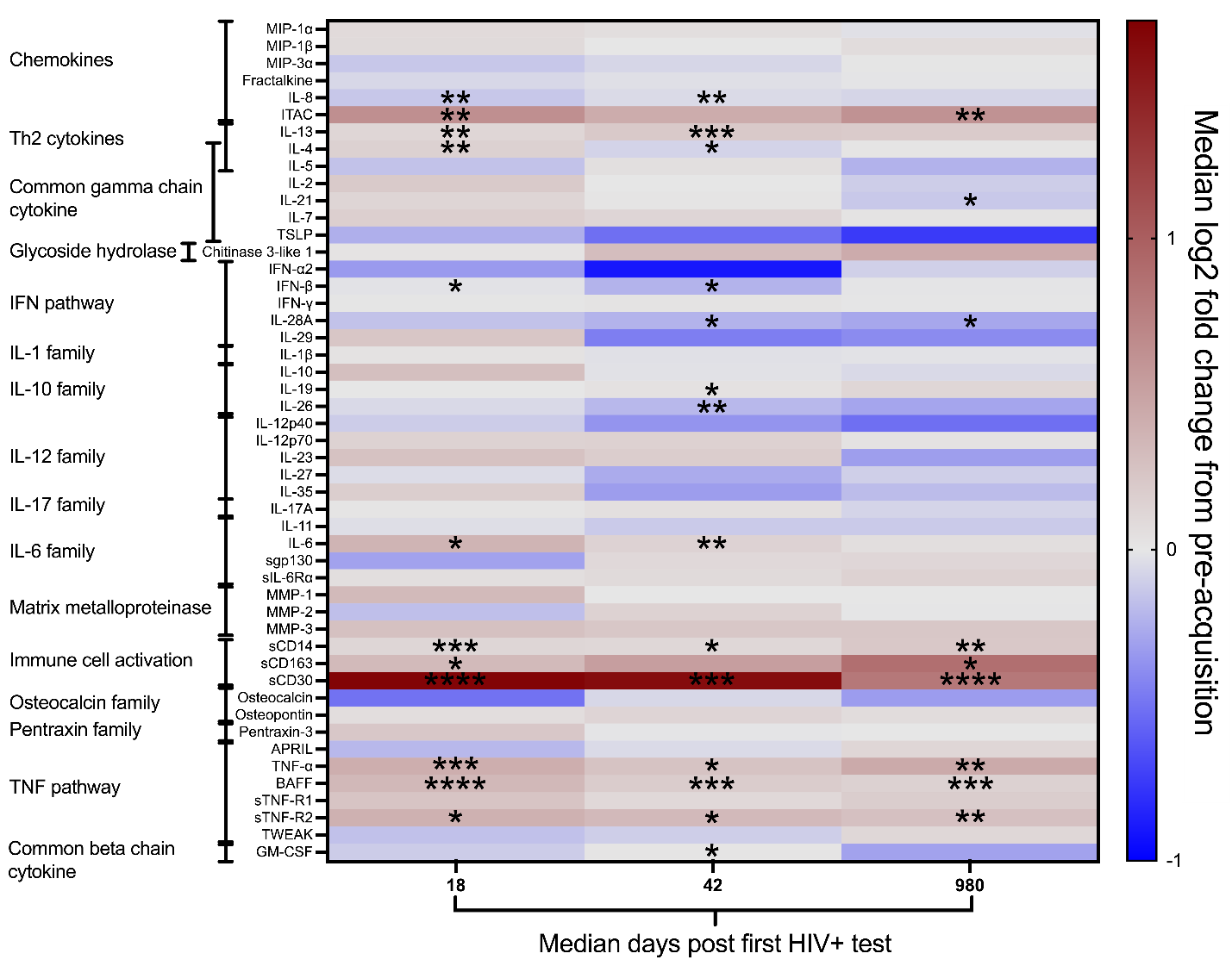


**Fig D.** Change in levels of soluble markers pre and post HIV acquisition. Heat map showing the median log2 fold change from pre-HIV acquisition at peak viral load (median day 18) setpoint viral load (median day 42), and chronic infection (median day 980) for each soluble marker measured across 22 individuals. *P < 0.05 **P < 0.01 *** P < 0.001 **** P < 0.0001.


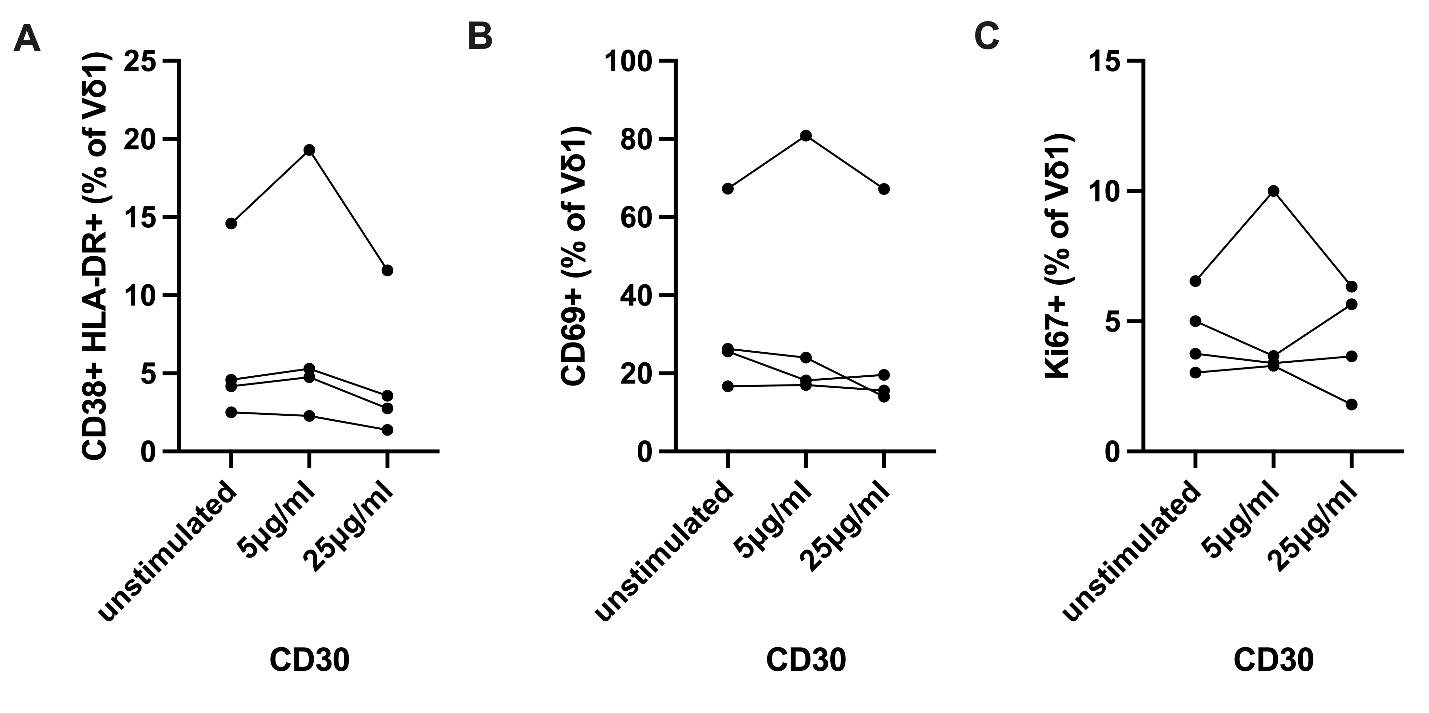


**Fig E.** PBMCs from 4 control individuals were culture in presence of 50 U/ml of IL-2 (Roche, Mannhein Germany) and CD30 (R&D Systems, Minneapolis, MN, USA) at 5 or 25 μg/ml for 2 days before evaluating the levels of CD38 and HLA-DR co-expression (A), CD69 (B), and Ki67 (C, anti-Ki67 BV750 clone B56, BD). The cells were fixed and permeabilized using FoxP3 / Transcription Factor Staining Buffer Set (Invitrogen, Carlsbad, CA, USA).


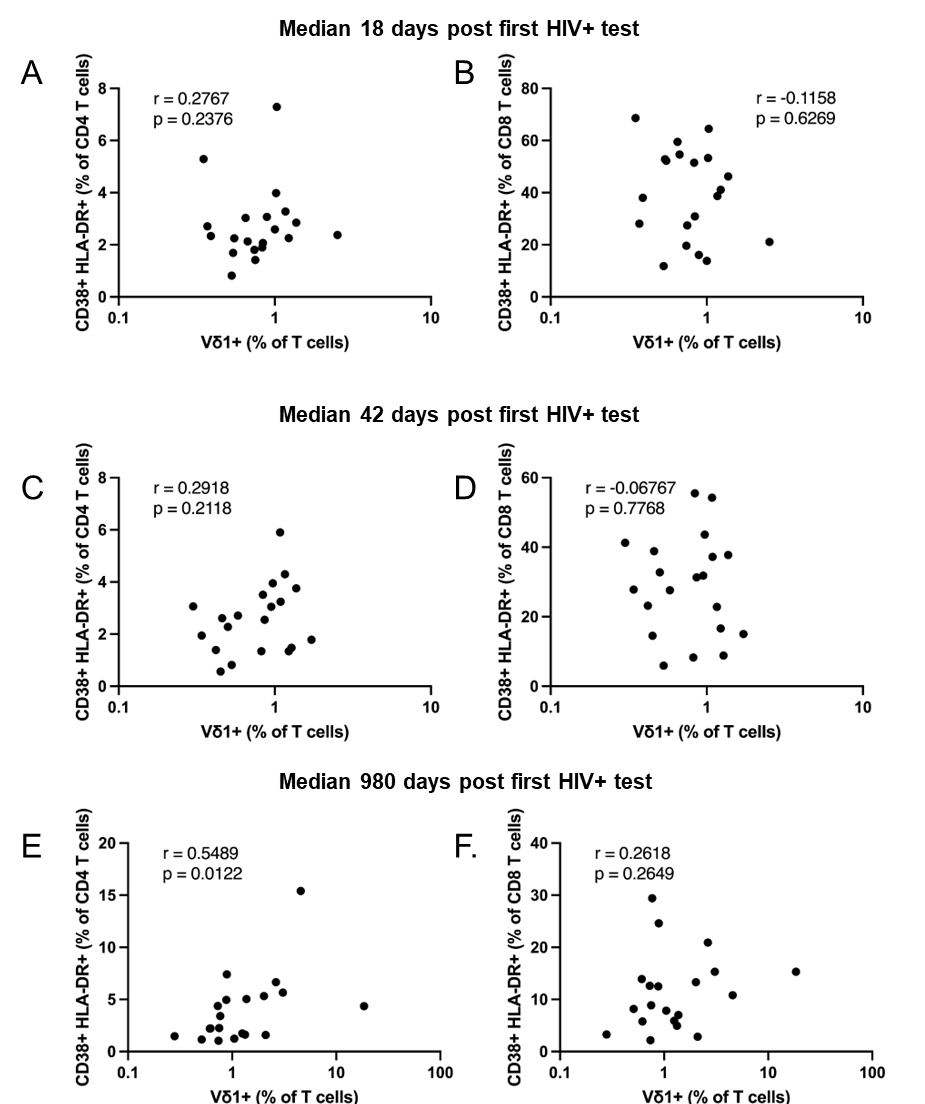


**Fig F.** Associations between CD38+ HLA-DR+ CD4 T cells or CD38+ HLA-DR+ CD8 T cells and Vδ1+ T cells frequency at median 18 (A-B), 42 (C-D), and 980 (E-F) days post first HIV-1+ test.


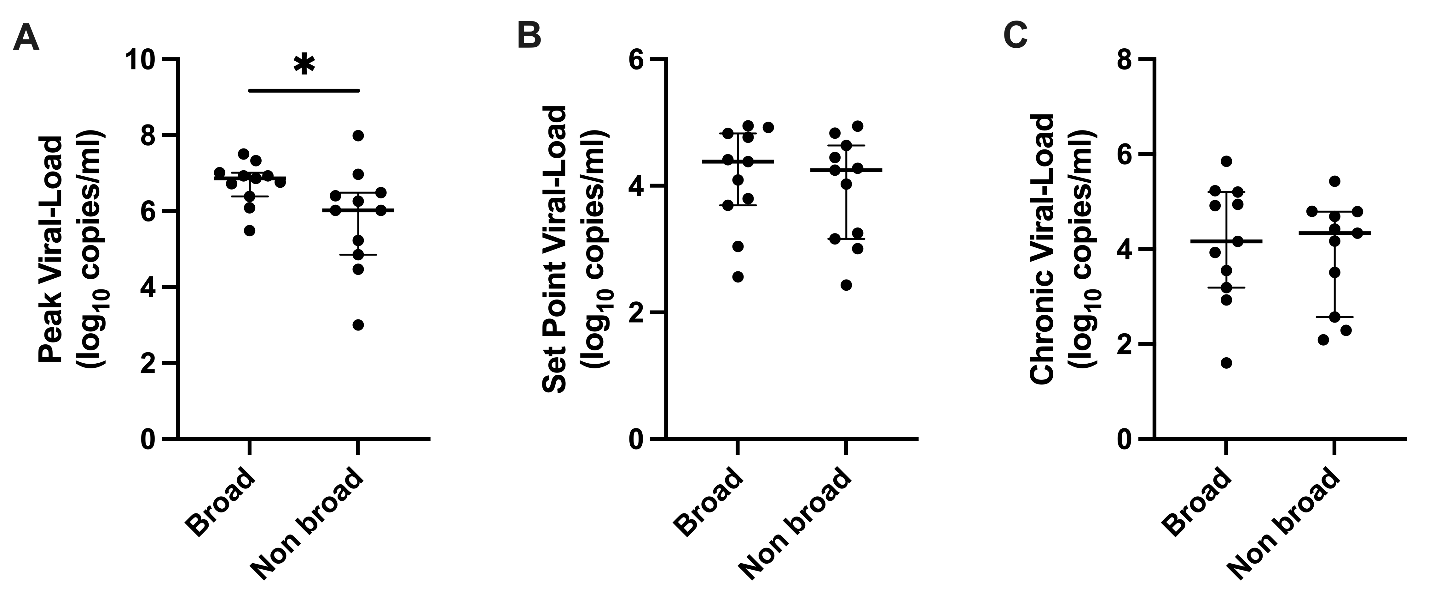


**Fig G.** Viral load comparisons between broad and non-broad neutralizers. Comparisons between the viral load in broad and non-broad neutralizers were made at peak VL (A), set-point VL (B), and chronic infection (C) using the Mann-Whitney test (A). *P < 0.05

**
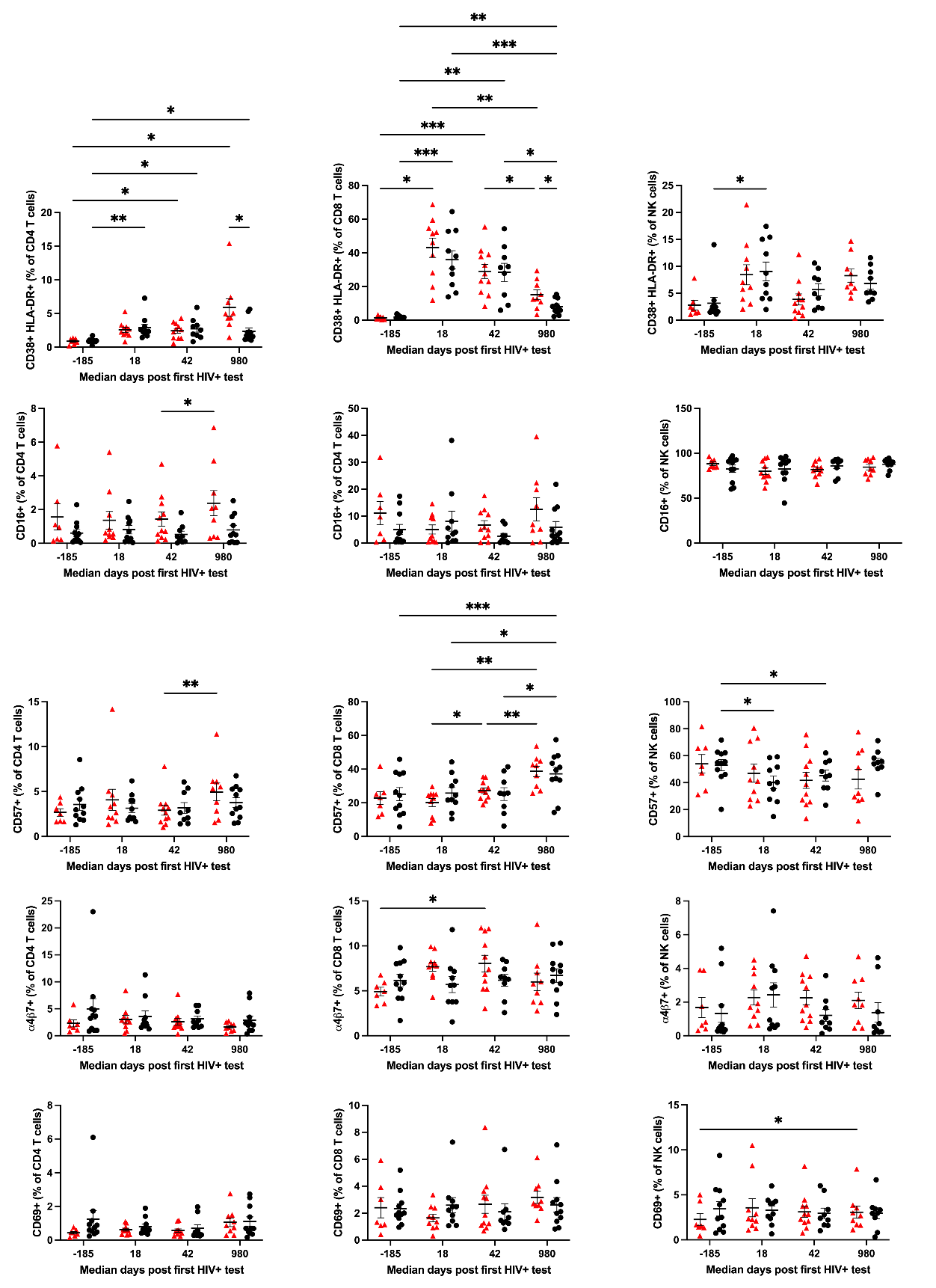
**

**Fig H.** Comparison between the phenotype of conventional CD4 and CD8 T cells as well as NK cells between broad and non-broad neutralizers pre- and post-HIV acquisition. Each data set is presented as the mean ± SEM. Broad and non-broad neutralizers were compared using a two-way mixed-effects ANOVA test. N= 7 and N=11 for broad and non-broad neutralizers respectively pre-HIV acquisition, N= 10 for broad and non-broad neutralizers at day 18 post first HIV+ test, N= 11 and N=9 for broad and non-broad neutralizers respectively at day 42 post first HIV+ test, N= 9 and N=11 for broad and non-broad neutralizers respectively at day 980 post first HIV+ test. *P < 0.05 **P < 0.01 and ***P < 0.001.


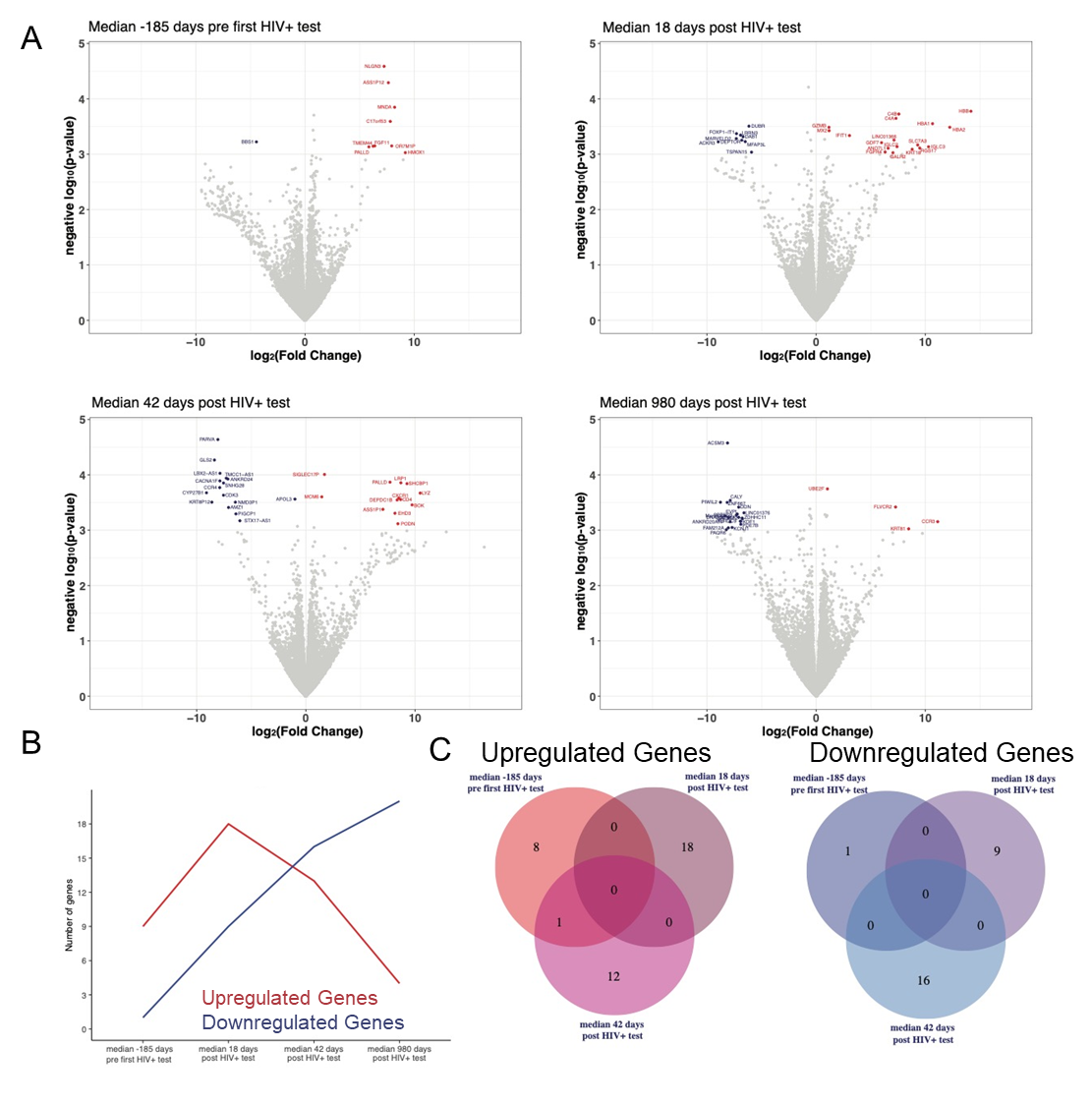


**Fig I.** Upregulated and downregulated genes in broad and non-broad neutralizers. Volcano plots depict upregulated (red) or downregulated (blue) genes in broad neutralizers compared to non-broad neutralizers at four timepoints including prior to the first positive HIV-1 test and median 18, 42, and 980 days post first HIV-1+ test (A). Highlighted genes have a -log_10_(p-value) ≥ 3 and a log_2_(Fold Change) of 1 or −1 (corresponding to P ≤ 0.001, and fold change of 2 or 1/2, in a generalized linear model). The temporal dynamics of the upregulated and downregulated genes in broad neutralizers compared to non-broad neutralizers are shown longitudinally post-HIV-1 acquisition (B). Shared and unshared differently expressed genes are highlighted as a Venn diagram (C). Increased genes are shown in shades of red and decreased genes are shown in shades of blue. There was no shared differently expressed genes at day 980 days post first HIV-1+ test.


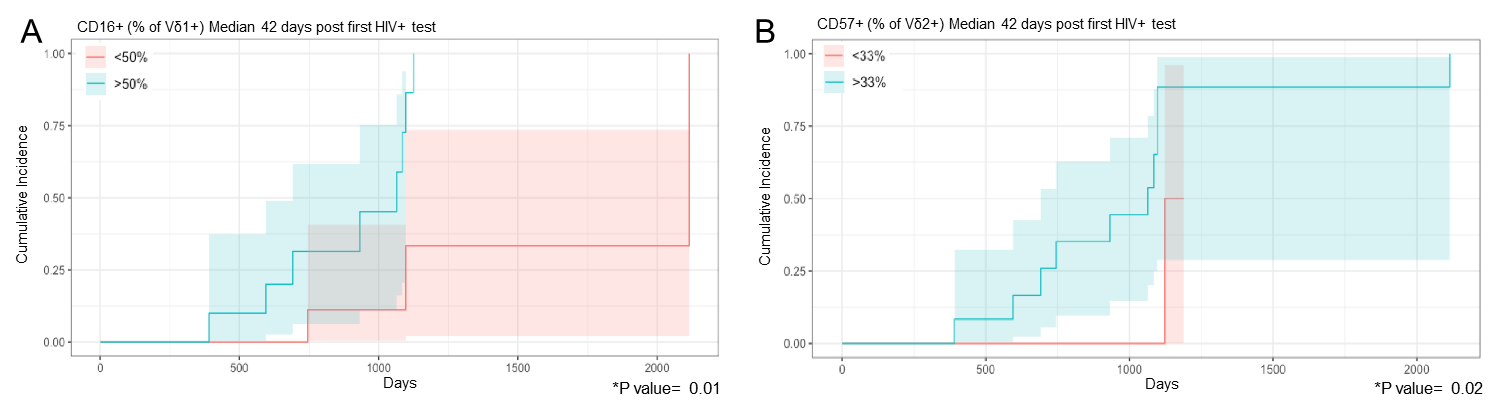


**Fig J.** Cumulative incidence curves show that CD16+ Vδ1 T cell expression level above the median (A) and above the first tertile for CD57+ Vδ2 T cells (B) 1 month post initial viremia significantly separated the participants that will achieve neutralization breadth during the study from those that did not. P values were determined by the LogRank test.


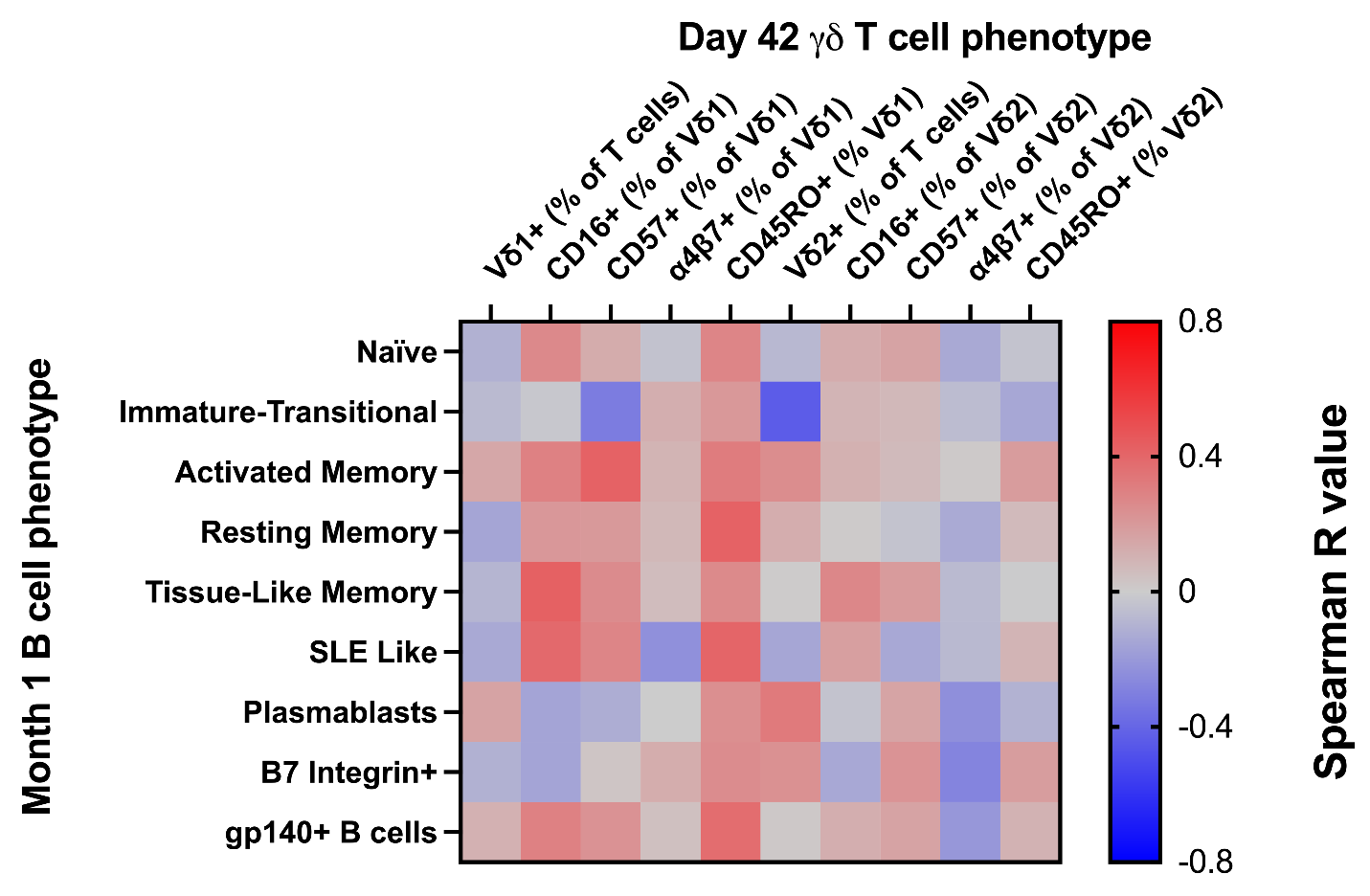


**Fig K.** Association between B cell phenotype 1 month post HIV+ test and and Vδ1+ and Vδ2+ T cell frequency and phenotype at median day 42 post HIV+ test. The heat map shows Spearman R values.
